# ‘There is no choice apart from antibiotics…’: Qualitative analysis of views on urinary infections in pregnancy and antimicrobial resistance

**DOI:** 10.1111/hex.13044

**Published:** 2020-02-29

**Authors:** Flavia Ghouri, Amelia Hollywood, Kath Ryan

**Affiliations:** ^1^ School of Pharmacy University of Reading Reading UK

**Keywords:** antibiotic resistance, antimicrobial drug resistance, interviews, pregnancy, qualitative research, self‐efficacy, urinary tract infection, women's health

## Abstract

**Background:**

Antimicrobial resistance (AMR) is a health risk as it can lead to life‐threatening infections. There has been a rise in resistant urinary tract infections (UTIs) which is the most common infection in pregnancy. This can be challenging in pregnancy due to the additional need to safeguard foetal development. The study's aim was to explore views about AMR in women who experienced UTIs in pregnancy.

**Design:**

Fifteen semi‐structured interviews were conducted in the UK and analysed using thematic analysis.

**Results:**

Results highlighted two themes: conceptualization of AMR and pregnancy as a deviation from the norm, with an overarching theme of ‘self‐efficacy’. Results show that participants were concerned about AMR but uncertain about the effect on society compared to individual's taking antibiotics and about completing antibiotic courses. Participants reported an unsparing use of antibiotics was justified in pregnancy, and behaviours like drinking adequate water were ineffective at preventing UTIs. In summary, women had low self‐efficacy regards tackling AMR and managing their health.

**Conclusion:**

Misconceptions about how AMR affects society vs the individual translated into viewing it as a future problem to be tackled by the health‐care sector. Consequently, AMR requires reconceptualization as a current problem requiring collective action. This research also indicates women endorse a biomedical model of UTIs in pregnancy which attributes resolving illness to interventions such as medicines, implying an automatic reliance on antibiotics. Subsequently, there is a need for self‐efficacy by focusing on a behavioural model which emphasizes behaviours for infection prevention, thus reducing the need for antibiotics.

## 1 INTRODUCTION

Antimicrobial resistance (AMR) is a global health threat and can result in serious or life‐threatening infections. Although AMR is a naturally occurring phenomenon, antibiotic use is its biggest driver because the use of these drugs causes positive selection of resistant bacteria. Antibiotic use can result in carriage of resistant bacteria by individuals for a period of several months to a year after completing a course.^1^ The bacteria can transfer to people in close contact and thus result in a spread of resistant infections. The transmission of resistant bacteria is particularly concerning in pregnancy as they may infect neonates during the birthing process. The choice of antibiotics in pregnancy can also be limited compared to the general population because of the risk of teratogenicity, which is the risk of harm to the developing foetus. Teratogenicity can exclude or restrict the use of antibiotics^2^ to specific trimesters; for example, trimethoprim is avoided in the first trimester due to a risk of neural tube defects and the risk of haemolysis precludes the use of nitrofurantoin at term.

Due to AMR, it is essential that antibiotics are used with a careful consideration of the benefits and risks. Antibiotics are the most commonly prescribed medicines in pregnancy,^3^ and urinary tract infections (UTIs) account for the majority of their use.^4^ In 2013, Public Health England established the English Surveillance Programme for antimicrobial utilization and resistance (ESPAUR) that monitors and publishes national data on AMR. The ESPAUR report from 2018 to 2019 shows that AMR in UTIs is established and an increasing burden in health care.^5^ The National Institute for Health and Care Excellence (NICE), who provide national care guidelines in the UK, published an update on its antimicrobial guideline for lower UTIs in October 2018 in response to this growing issue.^6^ Unlike respiratory infections, UTIs are caused by bacteria, and therefore, antibiotic treatment is usually required, especially if the infection is symptomatic. However, unlike the general population, antibiotic treatment is given in pregnancy if bacteria in the urine is detected even if the patient is asymptomatic.

A systematic review of interventions to prevent UTIs in pregnancy has shown that preventative hygiene behaviours are the only evidence‐based method associated with a reduced incidence of UTIs.^7^ Preventing the infection in the first place is therefore preferable to treatment through avoidance of behaviours associated with increased risk of UTIs, for example wiping from back to front after urination.^8^, ^9^ Although UTIs in pregnancy have been linked with risks such as pre‐term birth,^10^ there are also studies that suggest no such association.^11^, ^12^ A study analysing views from an online pregnancy forum shows that women associate UTIs with a high risk of negative outcomes, which might cause them to overlook the risks of AMR.^13^ A systematic review about public views of AMR has demonstrated that people might have incorrect scientific knowledge or apathy about AMR.^14^ The aim of the current study is to explore views on AMR in relation to UTIs by interviewing women who have experienced a UTI in pregnancy, and the research question is ‘how do women view AMR in relation to UTIs in pregnancy?’. The anticipated outcome of the study is to have an impact on the optimization of antibiotics for UTIs in pregnancy through an improved understanding of women's views about AMR.

## 2 METHOD

### 2.1 Design and procedure

This study used a qualitative design and consisted of 15 semi‐structured telephone interviews with women who had experienced a UTI in pregnancy. The inclusion criteria were women who were over 18 years old, resident in the UK and had experienced a UTI during pregnancy. The interviews mostly took place 2 years after the pregnancy where they experienced the UTI (the mode value for this data set was 2 years). All participants except one (participant four) took antibiotics for the treatment of the UTI. Participant four employed behavioural measures, such as drinking plenty of water, to resolve the UTI. The demographic characteristics of the participants can be seen in Table 1. Purposive sampling was used to recruit participants through advertisement of the study in online pregnancy forums (www.mumsnet.com and www.netmums.com) and social media (Twitter and Facebook posts). Interviews were conducted between July 2018 and January 2019. Most participants were recruited by advertising the study through the National Childbirth Trust social media account.

**Table 1:** Participant demographics

Participant number	Age (years)	Ethnicity	Education	Employment
P1	31	White	Degree	Full time
P2	18	White	GCSE	Not working
P3	31	White	Degree	Full time
P4	33	Other	Degree	Full time
P5	32	White	Degree	Full time
P6	35	White	Degree	Full time
P7	32	White	Degree	Not working
P8	38	White	Degree	Full time
P9	43	White	Degree	Part time
P10	31	White	Degree	Part time
P11	29	White	Degree	Not working
P12	43	White	A level	Full time
P13	27	White	A level	Not working
P14	31	White	Degree	Not working
P15	31	White	GCSE	Full time

The participant information sheet and consent form were emailed to women who expressed an interest in participating in the study. Participants were encouraged to read the participant information sheet and contact the researchers if they had any questions or concerns. The telephone interview date and time was arranged once the participants had returned the signed consent form. Interviews were conducted by the lead researcher about women's beliefs on prevention strategies for UTIs and antimicrobial resistance. The interview schedule included the following open questions:

- How was your experience of getting a UTI during pregnancy?
- How do you think UTIs impact pregnancy?
- What do you think about using antibiotics to treat UTIs during pregnancy?
- What do you think about antimicrobial resistance?
- What do you think about using alternatives to antibiotics to treat or prevent UTIs in pregnancy?

The interviews were recorded using an audio recorder to aid transcription. Interview recordings were transcribed verbatim for analysis by the lead researcher, and an honorary research assistant with all transcriptions double‐checked for accuracy. The average length of the interviews was 24.3 mins (SD ± 4.2) ranging from 18 to 29 minutes. Participants were gifted a £10 Amazon voucher at the end of the interview to thank them for their participation.

### 2.2 Data analysis

Data collection and analysis occurred concurrently to recognize saturation of themes and to guide when recruitment should be stopped. The data were organized into codes using NVivo 11^©^ and analysed using inductive thematic analysis.^15^ Thematic analysis is a flexible qualitative method and was chosen to allow the identification, analysis and interpretation of patterns in the data. Interview transcripts were read multiple times by the lead researcher to become familiar with the data and form detailed codes. The codes were further developed into themes by careful reflection of the patterns recognized in the data. Themes were reviewed, discussed and approved by all the authors.

### 2.3 Ethical approval

Participants were asked about their illness experience, so it was anticipated that there may be a risk of emotional distress. An information sheet was provided prior to the interview to notify participants of the topics that would be covered. They were also advised at the start of the interview that they were free to not answer any question if they were uncomfortable and could withdraw at any point during the interview. Documented consent was obtained from the participants prior to the interview. The study was reviewed and granted ethical approval by University of [Anonymised] Research and Ethics Committee (Ref. 17/30).

## 3 RESULTS

Inductive thematic analysis of the interviews with women who had experienced a UTI in pregnancy yielded two main themes: how women conceptualize AMR and how pregnancy causes a deviation from the norm in terms of antibiotic use. Transcending these themes was an overarching theme of self‐efficacy. The two themes highlight women's self‐efficacy in terms of what can be done in response to AMR and how they can manage their health with regards to UTIs. Quotes from the data have been used to illustrate the themes with a reference number indicating the order of participation (P1 = participant no. 1) and the trimester of pregnancy in which they experienced the infection.

### 3.1 Theme 1: Conceptualization of antimicrobial resistance (AMR)

Participants demonstrated a mixed understanding about AMR. Misconceptions that have already been identified through previous studies were also expressed by some women in this study. For example, several women thought that continual exposure to antibiotics makes the body resistant as opposed to the bacteria developing resistance and they indicated uncertainty in terms of how resistance is transferable. Some women, however, were aware that resistance is a characteristic of bacteria.

I am aware that if you overuse them [antibiotics], then they [antibiotics] will eventually stop, the body will stop working with them. (P15, 2nd trimester)

Well I assumed that it was an individual that built up resistance because they were given a lot of antibiotics and that eventually it stops working on that person. I'm not sure how it works if you've never taken antibiotics and then you need them. (P1, 1st trimester)

The drugs used to treat infections are becoming less effective because uhm, I mean, I am not a scientist, so I am probably not describing this properly, but the microbes within the infection are developing ways of overcoming the treatment. (P7, 3rd trimester)

Despite some misconceptions, all the participants except one were aware of AMR and recognized it as a health threat because of overuse of antibiotics. They were aware that AMR meant that antibiotics might not be effective against infections; however, most of the participants spoke about AMR as a distant phenomenon, as something that might happen ‘in the future’ as opposed to a current problem.

I think I've heard in the media that they've been overprescribed in the past and – and, we might end up at a point where some of us are resistant to uh, like, they won't help us. (P11, 1st trimester)

The most common solution cited by participants, in response to AMR, was to increase the public's understanding of the phenomenon. One participant mentioned the need for better diagnostics to optimize the use of antibiotics (P3), and two participants (P4 and P14) mentioned prevention as the primary way to avoid antibiotic use and tackle AMR.

I know it doesn't quite exist yet but sort of a definitive test to say, yes this is definitely a bacterial infection, yes we need antibiotics, and then later on down the line something to even tell you the best type of antibiotics so that you don't end up taking one course of antibiotic that your bug is resistant to and then needing a second course of another one. (P3, 2nd trimester)

I think we have to solve the problem in a more natural way for instance through increasing our immune system so this would be much more beneficial for future as well. (P4, 2nd trimester)

In summary, participants were aware of AMR but conceptualized it as a distant health threat. There was uncertainty about how people become infected with resistant bacteria even when they may not have used antibiotics themselves. Most participants were unsure about potential solutions but recognized a need for public awareness and suggested improved diagnostics and a focus on infection prevention to optimize antibiotic use.

### 3.2 Theme 2: Pregnancy as a deviation from the norm

Participants highlighted their pregnancy as an exceptional state, compared to the general population, when referring to their use of antibiotics because of the risks of UTIs. Most described themselves as ‘someone who does not like taking antibiotics’ but felt it was the safest option and this was also the view communicated to them by health‐care professionals.

I don't like taking antibiotics anyway, I don't – I'm not someone who takes antibiotics. (P10, 2nd trimester)

Um, I – I think this is where she [pharmacist] just said, if – you know, if the infection goes from your urinary tract into the womb that it, it could be very very serious, that it could – the thing that was the trigger, was she [pharmacist] said you know, it could go wrong very quickly. (P9, 1st trimester)

The reluctance to use antibiotics ranged from concern about side‐effects to an awareness about AMR, and many women expressed an interest in alternative therapies, such as probiotics. However, they recognized that antibiotics were the only effective treatment currently available and any new treatment would still be a concern due to unknown teratogenic risks.

It's a very good idea, anything that reduces the need for antibiotics. It depends what they are in some respects so if you're talking about probiotics or food supplements or something like that, that's one thing, but if it's a novel drug then you're always concerned about new drugs in pregnancy. (P3, 2nd trimester)

Most women were also aware of preventative hygiene behaviours but did not consider them effective.

Um well, you know, normal hygiene that everyone knows. Sort of wiping from front to back and general cleanliness – although I mean I suppose that doesn't make much difference. (P12, 3rd trimester)

Thus, although participants described a preference for avoiding antibiotics, they felt they did not have a choice because of the unacceptable risks of UTIs in pregnancy. Pregnancy was interpreted as a deviation from the norm, where an unsparing use of antibiotics was required and justified. Furthermore, hygiene behaviours, such as the direction of wiping the genitals, were considered to have little impact on prevention of UTIs.

### 3.3 Overarching theme: Self‐efficacy

Salient across both themes was an overarching theme that highlighted women's perceptions of self‐efficacy in terms of how AMR could be tackled and how they could manage their health in pregnancy. Self‐efficacy, as defined by Bandura,^16^ is ‘the belief in one's capabilities to organize and execute the sources of action required to manage prospective solutions’.

The first theme showed that most participants described conflict regarding how AMR could be tackled because although antibiotics cause problems, they ‘also save lives’ (P8) which causes a dilemma around how they can be used appropriately. Women also referred to conflicting messages about which behaviours they should adopt, particularly in response to finishing a course of antibiotics.

You get the odd media report saying that, you know, you shouldn't finish the course and your doctor's telling you to finish the course, so I think there is a lot of misinformation about resistance. (P3, 2nd trimester)

As a result, participants were unsure about how they could respond to AMR on an individual level and assigned the accountability for addressing the issue to health‐care professionals.

I would say there is a bit of an issue with GPs over prescribing antibiotics and there needs to be more awareness at the health‐care professional level. (P7, 3rd trimester)

The second theme described women's views about UTIs and showed that most participants perceived UTIs as a result of reduced immunity in pregnancy. They viewed the cause of the illness as outside their control and attributed pregnancy rather than individual behaviour to causing the infection.

I guess your immune system is slightly weakened isn't it, umm, so you are more susceptible…. (P12, 3rd trimester)

You're so susceptible when you're pregnant because your immune system seems to be so uhm… compromised when you're pregnant. (P1, 1st trimester)

Conflicting messages caused uncertainty and undermined women's confidence about what difference they could make thus resulting in low self‐efficacy in relation to tackling AMR. Similarly, perceptions of UTIs, where the role of behaviour was undermined, reduced women's self‐efficacy in managing their health through preventative hygiene behaviours. In summary, participants had low self‐efficacy with regard to managing personal and societal health.

## 4 DISCUSSION

This study qualitatively explored views on AMR in women who had experienced a UTI in pregnancy. The first theme demonstrates that participants were aware that antibiotics are overused at a population level, which can reduce their effectiveness and cause a health risk. However, misconceptions described by previous research,^17^, ^18^, ^19^ such as the body becoming resistant at an individual level, were still prevalent. The results revealed uncertainty due to conflicting messages from their doctor and the media regarding when and how antibiotics should be used. There was also greater understanding of AMR affecting the individual taking antibiotics in comparison with all sectors of society. Consequently, consistent with the findings of Hawkings et al,^20^ results from this research highlight that people prefer to delegate the responsibility of tackling AMR to health‐care professionals. A survey about public views of AMR by Carter et al^21^ reported that people did not consider AMR to be an important issue; however, in contrast, results from this study show that women were concerned about AMR but reported low self‐efficacy rather than apathy about the problem.

The second theme describes perceptions of pregnancy as a unique physiological state in which immunity is reduced and preventative behaviours have little impact. High perceived susceptibility during pregnancy was thought to result in an increased incidence of UTIs. At the same time, the severity of UTIs was also perceived to be greater compared to when not pregnant. Ogden et al^22^, ^23^ described a model where causes and solutions to illness are attributed to external and/or internal factors. The model highlights a coherence between the causes and solutions of illness whereby people expect illnesses to be resolved through external sources, such as medicines, if they believe the cause of the illness is external. The perceptions of women in this study were reflected in the biomedical model^24^ in which the cause of disease is attributed to medical or biological factors that were not necessarily under the control of the individual. The biomedical model therefore focuses on external interventions such as medicines sought for resolution of illness. Women's perceptions reflected this model and were combined with heightened perceived susceptibility and severity of UTIs. They also held medical advice in high esteem and therefore, to minimize any risks to their baby, reported an automatic reliance on antibiotics with reduced self‐efficacy in managing their own health. The dependence on antibiotics overrode behaviours, such as drinking adequate water, which could prevent illness and assist with symptom resolution in the case of an active infection. Overall, women considered antibiotics to be acceptable because they perceived pregnancy to be associated with high susceptibility and severity of UTIs. Future work could explore whether this positive perception of antibiotics also exists in relation to other infectious conditions that might occur in pregnancy.

The overarching theme present in the two main themes, conceptualization of AMR and pregnancy as a deviation from the norm, is self‐efficacy. Bandura's Social Cognition Theory^25^ about behaviour change outlines key factors that influence behaviour. Perceived self‐efficacy and outcome expectancies are two major constructs of the Social Cognition Theory. Self‐efficacy relates to an individual's sense of agency about a behaviour while outcome expectancies relate to beliefs about the consequences of the behaviour. Luszczynska and Schwarzer^26^ reviewed the Social Cognition Theory in the context of health behaviours and described how both these constructs can work in synchrony, which is seen in the present study. Women's outcome expectancy for preventative behaviours, such as drinking adequate water or the direction of wiping the genitals, was that they were ineffective. This outcome expectancy subsequently linked to low self‐efficacy in terms of managing their own health in pregnancy and avoiding antibiotics to conserve them for the communal good.

With regard to public health, the results from this study have implications for how antibiotics are viewed and utilized by the public, particularly in pregnancy. There is a need to re‐conceptualize AMR as a current, as opposed to a future, problem affecting society rather than just individuals, which requires action by both the public and health‐care professionals. This study proposes that this can be achieved by enhancing people's self‐efficacy through consistent messages about behaviours that are helpful to manage and reverse the risks of AMR. Bandura^27^ has described sources of information (mastery experiences, vicarious experiences, verbal persuasion, and physiological and affective states) that enhance self‐efficacy. Future research on health behaviours linked with AMR may find it useful to explore the role of these sources in enhancing self‐efficacy for behaviour change. A consolidated approach, where infection prevention is prioritized and where people are empowered, is also endorsed by the World Health Organization (WHO).^28^ The WHO global action plan on antimicrobial resistance emphasizes that the response to AMR needs to be through a focus on infection prevention, along with engagement and empowerment of health‐care professionals and the public, across all sectors of society. Specifically, in pregnancy, there is scope for health‐care professionals to develop women's perceptions of self‐efficacy by highlighting preventative behaviours and linking them as a means of minimizing antibiotics for UTIs in response to AMR. This shift from a biomedical model to a behavioural model could lead to better and sustained health outcomes.^29^

The study provides unique insight into perceptions of AMR by focusing on women with personal experience of UTIs in pregnancy. Conducting an interview study had the advantage of exploring the issue in‐depth by asking participants to expand or clarify their views through conversation. One of the main limitations of the study was that it focused on a small subset of the population using purposive sampling, which reduces generalizability. As the sample consisted predominantly of women who were White British, the views in this study may not be representative of women whose demographics differ significantly from the participants and need to be interpreted within this context. Future work will focus on exploring the views of health‐care professionals to provide a comprehensive understanding of AMR and UTIs in pregnancy.

## 5 CONCLUSION

In conclusion, women recognize the risks of AMR but demonstrated low self‐efficacy and perceived control of UTIs in pregnancy. There is a need to re‐conceptualize AMR and provide a consistent message to avoid uncertainties. Women might require reassurance specifically in pregnancy to feel confident about their ability to manage their own health with an emphasis on behaviours that can prevent UTIs to reduce the need for antibiotics.

## CONFLICT OF INTEREST

There are no conflicting interests.

## Data Availability

The data that support the findings of this study are available from the corresponding author upon reasonable request.
